# How Do Patients Rate Their Radiation Oncologists in the Modern Era: An Analysis of Vitals.com

**DOI:** 10.7759/cureus.3312

**Published:** 2018-09-17

**Authors:** Simrath Randhawa, Asim Viqar, Julia Strother, Arpan V Prabhu, Fen Xia, Dwight Heron, Sushil Beriwal

**Affiliations:** 1 Radiation Oncology, University of Pittsburgh Cancer Institute, Pittsburgh, USA; 2 Medicine, Perelman School of Medicine, University of Pennsylvania, Pittsburgh, USA; 3 Medicine, Frank H. Netter Md School of Medicine at Quinnipiac University, North Haven, USA; 4 Radiation Oncology, Winthrop P. Rockefeller Cancer Institute, University of Arkansas for Medical Sciences, Little Rock, USA; 5 Radiation Oncology, University of Pittsburgh Medical Center, Pittsburgh, USA; 6 Radiation Oncology, University of Pittsburgh Cancer Institute, University of Pittsburgh Medical Center, Pittsburgh, USA

**Keywords:** radiation oncologist, overall rating, physician related factors, online ratings, vitals.com, radiation oncology, digital identity, physician-review website, patient satisfaction

## Abstract

Introduction

The popularity of online physician rating websites has risen substantially. These third-party sites have the potential to significantly influence patients’ perception of their healthcare providers. The purpose of this study was to evaluate online ratings of U.S. radiation oncologists (ROs) on Vitals.com, one of the most popular physician rating websites, and the variables that most significantly affect patients’ overall rating (OR) of their ROs.

Methods

The Centers for Medicare and Medicaid Physician Comparable Downloadable File was analyzed to obtain data on all self-identified ROs in the U.S. and Puerto Rico. Patient Review Satisfaction Scores (PRSS) that ranged from one (poor) to five (excellent) for the following variables were recorded: OR, accurate diagnosis, spending appropriate time with patients, ease of appointment, courteous staff, bedside manner, follow-up after visit, promptness, and wait time. Associations among these factors were assessed.

Results

Of 4,443 self-identifying Medicare-accepting ROs, 1,797 (40.4%) ROs who had at least one OR rating and at least one written comment were included in this study. The ROs’ mean OR was 4.34 ± 0.2 (median 4; 30% received a score of 5; 78% received a score greater than 4). OR was found to have a strong correlation with accuracy of diagnosis (*r* = 0.69), bedside manner (*r* = 0.71), and spends appropriate time with patients (*r* = 0.69). With the exception of the number of ratings (*p* = 0.07), physicians with over 10 years of experience showed statistically significant differences in how much better they scored in each of the variables compared to those with less than 10 years of experience (*p* < 0.01 for each characteristic). Significant differences in OR were also observed between ROs whose wait times exceeded 20 minutes compared to those with wait times less than 10 minutes (*p* < 0.01) for all internal and external metrics except for the number of ratings (*p* = 0.42) and number of reviews (*p* = 0.88)

Conclusion

Patients are providing high ratings for their ROs on Vitals.com and are more frequently recommending them to friends and family. Given the rise in popularity of third-party physician rating sites, it is important for ROs to understand the various factors that may influence their online ratings.

## Introduction

With the movement toward increased patient participation in decision making and greater overall healthcare transparency, third-party physician-review websites (PRWs) have grown in popularity. The proportion of Americans who utilize the Internet to gather health-related information has tripled within the past decade [1-4]. Following the advent of PRWs, patients increasingly seek information regarding the quality of the physicians available to inform their decisions about which providers to choose across virtually any medical specialty. About 90% of physicians now have professional information available online through PRWs [5].

PRWs, therefore, represent a unique opportunity to characterize how patients feel about their physicians and identify the variables that most significantly contribute to patients’ overall perception of their healthcare providers. Previous work by our group showed that patient satisfaction scores for radiation oncologists (ROs) were very strong on Healthgrades.com, and most patients leaving reviews were likely to recommend their own ROs to their friends and family [6].

The purpose of this study was to evaluate physician ratings on Vitals.com, a PRW that is among the most frequently visited across all specialties and is estimated to receive roughly 500,000 users a year* *[7]. The reviews analyzed in this study were explored specifically in the context of radiation oncology due to the limited body of research surrounding this medical specialty.

## Materials and methods

This study was IRB exempt because it utilized publicly available federal databases and web-accessible data sources.

Study population

A list of ROs was created through the Centers for Medicare and Medicaid Services (CMS) Physician Comparable Downloadable File (PCNDF) [8]. Data obtained through the PCNDF was accessed and cleaned on September 23, 2016 using National Provider Identifier (NPI) numbers. The PCDNF serves as a representation of 91% of physicians practicing in the U.S. as it includes all who are enrolled in Medicare fee-for-service. This allows it to function as a comprehensive sample of physicians in the U.S [9-10].** **

The PCNDF list of radiation oncologists was analyzed using Python and Pandas, a program that functions as an open-source library [11]. From this PCNDF list, information regarding first name, last name, NPI number, gender, degree type (MD or DO), medical school graduation year, and the city and state of physicians’ practices were obtained. 

Data collection

All physician review data was obtained from Vitals.com, where ratings range from one to five (1 = poor; 2 = fair; 3 = good; 4 = great; 5 = excellent). The minimum requirement for patients to rate a physician on Vitals.com is a global measure of patient satisfaction signified on the website as overall rating (OR) [12]. Patients may also rate a physician in a number of categories related to professionalism and quality. These metrics include the following: “accurate diagnosis,” “spends time with me,” “ease of appointment,” “courteous staff,” “bedside manner,” “follow-up after visit,” and “promptness.” Patients also have the option to include the approximate wait time (a drop-down menu allows for the selection of times based on five-minute increments).

These descriptive factors were classified as either office-related factors or physician-related factors.“Courteous staff” and “ease of appointment” were classified as office-related factors, and physician-related factors included “accurate diagnosis,” “spends time with me,” “promptness,” “bedside manner,” and “follow-up after visit.” 

Inclusion criteria for this study required that an RO have at least one rating for OR and at least one written comment. The following data were collected, if available, for each RO that met these inclusion criteria: years of experience, primary state of practice, OR, ratings in the categories related to professionalism and quality described above, and the total number of ratings and number of reviews.

Data analysis

Summary Statistics

Responses were summarized through the calculation of standard summary statistics.

Mean LTR Correlation with Various Factors

Spearman correlation coefficients were computed, with a value between 0.81 and 1.00 indicating very strong agreement and a value between 0.61 and 0.80 indicating strong agreement. Any value lower than 0.61 was not considered to be of any significant agreement. These coefficients were used to calculate the relationship between OR and each metric, years of experience, and the number of patient reviews.

Mann-Whitney Test

Mean OR scores were computed for each gender and compared through the Mann-Whitney Test. Mean OR scores, physician-related factors, and office-related factors were all compared among physicians with over 10 years of experience and those with less than 10 years of experience. Differences in mean OR scores, physician-related factors, and office-related factors were also determined for ROs whose wait time was under 10 minutes and those whose wait time exceeded 20 minutes.

Kruskal-Wallis Test

The Kruskal-Wallis test was used to compare the calculated mean OR score of each geographical region. The US Census Bureau’s geographical definitions were utilized to appropriately designate US regions per the following: 1 = Northeast, 2 = Southeast, 3 = Midwest, 4 = West [13].

Internal Validity

The Cronbach alpha statistic was calculated for individual office-related factors and physician-related factors to evaluate how effectively these variables assessed the same underlying construct.

## Results

Data collection and summary statistics

**Figure 1:**
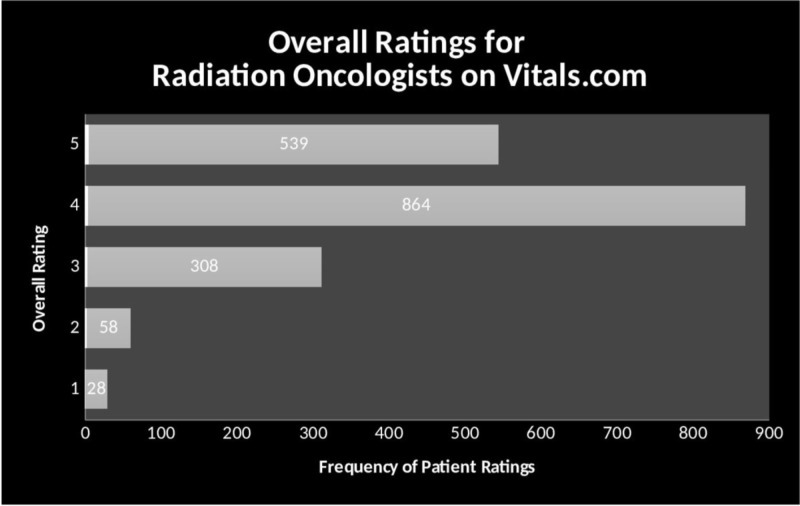
Overall Ratings for Radiation Oncologists in Vitals.com

Out of 4,443 self-identifying Medicare-accepting ROs, 1,797 (40.4%) ROs had at least one rating for OR and at least one written comment for inclusion in this study. Exactly 539 physicians (30%) received the highest score possible (5.0), and 1,403 (78%) received a score of very good or above (>4.0) (Figure 1).

The mean number of patient reviews was 4.50 ± 0.9 (mean ± standard deviation) (Table 1). The range for the number of patient ratings per physician spanned from 1 to 583 with a mean of 8.48 ratings (Table 2). Only 5% of wait times exceeded 30 minutes. More than half of ROs received more than one review, while 1,678 (93%) received more than one rating. The mean OR ranged from very good to excellent (4.34 ± 0.02) (Table 2). 

**Table 1:** Summary of All 1,797 Physicians

Years since graduation	24.5 ± 0.2, median: 25
0-10 years	11.0% (192)
11-20 years	26.4% (469)
21-30 years	35.3% (628)
31+ years	27.9% (495)
Geographic region	
Northeast	20.8% (374)
Southeast	33.4% (593)
Midwest	21.7% (390)
West	23.7% (426)
Number of patient reviews	4.50 ± 0.9 (median 2, range 1-242)

Mean OR Correlation with Variables

OR showed a strong correlation with accuracy of diagnosis (*r* = 0.69), bedside manner (*r* = 0.71), and spends appropriate time with patients(*r* = 0.69) (Table 2). OR showed a fair level of correlation with the following metrics: ease of scheduling appointments (*r* = 0.56), promptness (*r* = 0.60), staff friendliness (*r* = 0.58), and whether appropriate follow-up was given (*r* = 0.47). OR showed a weak negative correlation with wait time (*r* = -0.36). No correlation was seen between OR and the number of patient reviews (*r* = -0.01).

**Table 2:** Mean Score for Survey Items and Correlations with Overall Rating Bold values indicate statistical significance * Gender was assigned on a scale of Male = 1; Female = 2 *Graduation year was assigned as follows: <10 years ago = 1. 10-20 years ago = 2. 21-30 years ago = 3. 31+ years ago = 4 *Academic status was assigned as the following: Nonacademic: 1; Academic: 2 *Location assigned as following: Northeast = 1 Southeast = 2 Midwest = 3 West = 4

	*n *(%)	Mean	Correlation with overall rating
Overall rating	1797(100)	4.34±0.02	n/a
*Office-related factors*			
Ease of scheduling appointments	1773(99)	4.60±0.02	0.56
Staff friendliness	1776(99)	4.64±0.02	0.58
*Physician-related factors*			
Spends appropriate amount of time with patient	1772(99)	4.53±0.02	0.69
Promptness	1772(99)	4.50±0.02	0.60
Bedside manner	1772(99)	4.61±0.9	0.71
Accuracy of diagnosis	1771(99)	4.59±0.02	0.69
Appropriate follow-up	1759(98)	4.53±0.02	0.47
Average RO wait time (min)	1589(89)	12.5±0.2	-0.36
Number of patient ratings	1797(100)	8.48±0.5	0.02
Number of patient reviews	1797(100)	4.50 ±0.9	-0.01
Graduation year*	1777(99)	24.5±0.2	0.01
Location*	1791(100)	2.53±0.2	n/a

Mann-Whitney Test

OR showed no association with ROs’ years since graduation (*p* = 0.12) or gender (*p* = 0.08). There was a statistically significant difference between the OR for ROs with less than 10 years of experience (4.25 ± 0.02) and ROs with more than 10 years of experience (4.47 ± 0.02) (*p* < 0.01). With the exception of number of ratings (*p* =0.07), ROs with over 10 years of experience showed statistically significant higher scores in all of the other variables in this study compared to ROs with less than 10 years of experience (*p* <0.01 for each of these variables).

Furthermore, a statistically significant difference in OR was found between the 644 physicians whose wait time was under 10 minutes (4.46 ± 0.01) and the 248 physicians whose wait time exceeded 20 minutes (4.06 ± 0.04) (*p *< 0.01). Significant differences were also observed between these two groups of ROs in each of the internal and external metrics considered (p < 0.01) except for the number of ratings (*p* = 0.42) and number of reviews (*p* = 0.88). Having more than 10 reviews, however, was not associated with significant differences in the OR (4.36 ± 0.02 vs. 4.41 ± 0.02, *p* < 0.07).

Kruskal-Wallis Test

Mean OR did not show a statistically significant association (*p *< 0.08) with any geographical region.

## Discussion

In our study, we manually searched for self-reported patient data from a popular PRW and observed the majority of patient satisfaction scores for ROs to be in the range of great to excellent. These findings are consistent with our previous work regarding high patient satisfaction with ROs on Healthgrades [6], patient satisfaction literature examining hand surgeon ratings on three PRWs across the country [14], and literature on primary care physicians, medical specialists, surgical specialists, and obstetricians and gynecologists who practice in Virginia [1]. Given the current popularity of PRWs, the findings of this study are directly relevant to patients and ROs and can be viewed as additional data in the growing literature surrounding patient satisfaction. 

Vitals.com is among the two most commonly searched PRWs in the United States [7]. In our study, the OR of ROs showed the greatest correlation withbedside manner, accuracy of diagnosis, and spends appropriate time with patients, achieving strong levels of correlation with all three factors (Table 2). Our work aligns with previous findings that suggest that physician-related factors show the most direct relationship with the OR of ROs and oncologists [6,15-16]. 

Wait time was not significantly correlated with OR, in contrast with previous work highlighting the impact of RO wait time on patient satisfaction with their physicians [17-20]. Previous research has indicated that patient complaints over professionalism were more prevalent than complaints over wait time [21,22]. Matthews et al. found that physicians-related factors like interpersonal skills, coordination of care, and timeliness of care shaped patients' satisfaction with their wait times [23]. Despite the lack of a significant correlation between wait time and a lower OR in this study, lower scores in physician-related factors like bedside manner  (*r* = 0.71), accuracy of diagnosis (*r* = 0.69), and spends appropriate time with patients (*r* = 0.69) did show a strong association with a lower OR (Table 2). 

Interestingly, while previous work has shown no relationship between ROs' experience level and higher online ratings [6], our study showed that ROs with more clinical experience (more than 10 years versus less than 10 years of experience) had a significantly higher OR. Other research has highlighted the opposite, including a study by Gao et al., that shows that younger physicians (those who had graduated from medical school after 2000) had higher ratings than their older counterparts [1]. These differences could be partly attributed to the demographics of the patient populations that are more actively using PRWs and would be an interesting area for future study.

The use of PRWs by patients should encourage ROs to increase and improve their online presence [10]. Through blogs or personal webpages, ROs and other physicians can also adapt appropriate education materials to encourage patient engagement [24-28]. There are also greater opportunities for ROs to engage with the public through social media such as Twitter [29]. Interestingly, the results of this study showed no significant difference in the OR for ROs with more than 10 ratings versus those with less than 10 ratings, potentially demonstrating that ROs yet to establish an online presence will not be at an initial comparative disadvantage to ROs with greater clout when they establish an online identity on PRWs.

This study had limitations, as the use of Vitals.com creates sample bias as it is a voluntary patient-generated review site. Previous studies suggest that physicians with a lower quality performance tend to have a greater chance of being rated online, while physicians with higher quality performance tend to be less likely to receive online ratings and attention [30].Also, the authenticity of online reviews must be considered, as the anonymity that is granted to patients on such sites has the potential to be abused.The only requirement for leaving a review on Vitals.com is having an email account, which is kept anonymous. Demographic factors were not available for patients, which limits our ability to validate patients’ identities. It is possible that physician self-promotion may have had an influence on the findings in this study. Nevertheless, we believe that the data presented here illustrates the role of online rating websites in better understanding patient satisfaction.

## Conclusions

Patients are providing high ratings for their ROs on Vitals.com and are more frequently recommending them to friends and family. Given the rise in popularity of third-party physician rating sites, it is important for ROs to understand the various factors that may influence their online ratings.

## References

[1] Gao GG, McCullough JS, Agarwal R, Jha AK (2012). A changing landscape of physician quality reporting: analysis of patients' online ratings of their physicians over a 5-year period. J Med Internet Res.

[2] Harris KM, Buntin MB (2018). Choosing a health care provider: the role of quality information. Robert Woods Foundation.

[3] Shahian DM, Normand SL, Torchiana DF, Lewis SM, Pastore JO, Kuntz RE, Dreyer PI (2001). Cardiac surgery report cards: comprehensive review and statistical critique. Ann Thorac Surg.

[4] Fox S (2018). The social life of health information. Washington, DC: Pew Internet & American Life Project.

[5] Mostaghimi A, Crotty BH, Landon BE (2010). The availability and nature of physician information on the internet. J Gen Intern Med.

[6] Prabhu AV, Randhawa S, Clump D, Heron DE, Beriwal S (2018). What do patients think about their radiation oncologists? An assessment of online patient reviews on healthgrades.

[7] Kadry B, Chu F, Kadry B, Gammas D, Macario A (2011). Analysis of 4999 online physician ratings indicates that most patients give physicians a favorable rating. J Med Internet Res.

[8] (2016). Center for Medicare & Medicaid Physician Compare National Downloadable File Dataset. https://data.medicare.gov/Physician-Compare/Physician-Compare-National-Downloadable-File/mj5m-pzi6/data.

[9] Shartzer A, Zuckerman R, McDowell A, Kronick R (2018). Access to physicians’ services for Medicare beneficiaries. US Department of Health and Human Services.

[10] Prabhu AV, Kim C, De Guzman E (2017). Reputation management and content control: an analysis of radiation oncologists' digital identities. Int J Radiat Oncol Biol Phys.

[11] (2016). Python. http://www.python.org.

[12] (2018). Vitals.com. http://vitals.com.

[13] Bureau U. 2010 Census (2017). 2010 Census: apportionment data map. Commerce.

[14] Trehan SK, DeFrancesco CJ, Nguyen JT, Charalel RA, Daluiski A (2016). Online patient ratings of hand surgeons. J Hand Surg Am.

[15] Thomas S, Glynne-Jones R, Chai I (1997). Is it worth the wait? A survey of patients' satisfaction with an oncology outpatient clinic. Eur J Cancer Care.

[16] Geinitz H, Marten-Mittag B, Schafer C (2012). Patient satisfaction during radiation therapy: correlates and patient suggestions. Strahlenther Onkol.

[17] Gesell S, Gregory N (2004). Identifying priority actions for improving patient satisfaction with outpatient cancer care. J Nurs Care Qual.

[18] Gourdji I, McVey L, Loiselle C (2003). Patients' satisfaction and important ratings of quality in an outpatient oncology center. J Nurs Care Qual.

[19] Famiglietti R, Neal E, Edwards T, Allen P, Buchholz T (2013). Determinants of patient satisfaction during receipt of radiation therapy. Int J Radiat Oncol Biol Phys.

[20] Sandoval G, Brown A, Sullivan T, Green E (2006). Factors that influence cancer patients’ overall perceptions of the quality of care. Int J Qual Health Care.

[21] Salazar G, Quencer K, Aran S, Abujudeh H (2013). Patient satisfaction in radiology: qualitative analysis of written complaints generated over a 10-year period in an academic medical center. J Am Coll Radiol.

[22] Doshi AM, Somberg M, Rosenkrantz AB (2016). Factors influencing patients' perspectives of radiology imaging centers: evaluation using an online social media ratings website. J Am Coll Radiol.

[23] Matthews M, Ryan D, Bulman D (2015). What does satisfaction with wait times mean to cancer patients?. BMC Cancer.

[24] Prabhu AV, Hansberry DR, Agarwal N, Clump DA, Heron DE (2016). Radiation oncology and online patient education materials: deviating from NIH and AMA recommendations. Int J Radiat Oncol Biol Phys.

[25] Prabhu AV, Donovan AL, Crihalmeanu T (2018). Radiology online patient education materials provided by major university hospitals: do they conform to NIH and AMA guidelines?. Curr Probl Diagn Radiol.

[26] Prabhu AV, Crihalmeanu T, Hansberry DR (2017). Online palliative care and oncology patient education resources through Google: do they meet national health literacy recommendations?. Pract Radiat Oncol.

[27] Prabhu AV, Kim C, Crihalmeanu T, Hansberry DR, Agarwal N, DeFrances MC, Trejo Bittar HE (2017). An online readability analysis of pathology-related patient education articles: an opportunity for pathologists to educate patients. Hum Pathol.

[28] Hansberry DR, Ayyaswami V, Sood A, Prabhu AV, Agarwal N, Deshmukh SP (2017). Abdominal imaging and patient education resources: enhancing the radiologist-patient relationship through improved communication. Abdom Radiol (NY).

[29] Thomas J, Prabhu AV, Heron DE, Beriwal S (2018). Twitter and brachytherapy: an analysis of "tweets" over six years by patients and health care professionals. Brachytherapy.

[30] Gao GD, Greenwood BN, Agarwal R, McCullough JS (2015). Vocal minority and silent majority: how do online ratings reflect population perceptions of quality. MIS Quarterly.

